# Addressing the Persistent Social Challenges of Very Preterm Birth

**DOI:** 10.1111/ppe.13152

**Published:** 2024-12-10

**Authors:** Marina Mendonça

**Affiliations:** ^1^ Department of Psychology University of Warwick Coventry UK

The survival rates of individuals born very preterm (VP, < 32 weeks gestation) or with very low birth weight (VLBW, < 1500 g) have increased dramatically since the 1970s and 1980s, primarily due to significant, advancements in neonatal intensive care. As the first major generation of these survivors reaches established adulthood, understanding their long‐term outcomes becomes increasingly critical for healthcare providers and policymakers. Research has consistently demonstrated that children born VP/VLBW may experience an array of challenges, including increased risk for disabilities, lower cognitive abilities, impaired executive functions, mental health problems and social difficulties that may persist into adulthood [1]. These sequelae are frequently comorbid, resulting in complex and intersecting needs. Although most individuals born preterm lead healthy and well‐adjusted lives, meta‐analyses indicate that VP/VLBW birth may result in greater disadvantages regarding personal life chances [2, 3] in adulthood. For example, young adults born VP/VLBW, on average, tend to leave school with lower qualifications, are less likely to be involved in romantic partnerships and to have children themselves compared to their peers born at term.

In this issue of *Paediatric and Perinatal Epidemiology*, Gonen and colleagues [4] make an important contribution to our understanding of the long‐term impact of VP/VLBW birth by investigating social relationships with parents, partners and peers from age 26 to 34. More precisely, the authors investigated whether differences between VP/VLBW and term‐born adults in these relationships persist or narrow during the transition from emerging to established adulthood. Using a prospective whole‐population birth cohort study from South Bavaria (Germany) with a matched term‐born control group, the results of this study show a persistent gap in partner and peer relationships, with VP/VLBW‐born adults rating these relationships lower than their term‐born counterparts. Importantly, the quality of relationships with parents was not different between VP/VLBW‐ and term‐born adults. These findings provide compelling evidence that perinatal life events exert long‐term impacts on socio‐emotional functioning while contradicting historical concerns about the long‐term negative effects of early separation during neonatal intensive care on parent–child relationships. Furthermore, these findings support results from previous meta‐analyses [3, 5] that compared social outcomes across different age cohorts, show a relational gap between VP/VLBW‐ and term‐born individuals. The current study provides now longitudinal evidence of this gap showing that the social impact of very preterm birth extends well into adulthood.

The importance of close and meaningful relationships cannot be overstated. Building a supportive peer network, establishing healthy romantic relationships and maintaining strong ties with the family of origin are key developmental tasks of young adulthood that have been associated with beneficial health and well‐being [6] outcomes. These relationships may also set the foundation for later relationship success, including starting a family. Given their role in human development and health, understanding how these relationships evolve in individuals born VP/VLBW as they progress through adulthood is crucial.

In this study, both VP/VLBW‐ and term‐born groups show similar patterns of relationship development, with partner and peer relationships improving from emerging to established adulthood, while parent–child relationships naturally decrease during this transition. This parallel developmental trajectory suggests that VP/VLBW adults follow typical patterns of social development, albeit with persistent differences in peer and romantic domains. A notable finding is the robustness of parent–child relationships in adults born VP/VLBW. This resilience may reflect the increased parental investment often seen in families of preterm children [7], suggesting protective family dynamics. However, it is also important to further investigate whether the above‐mentioned persistent differences are caused by physical or cognitive problems or by different or overprotective parenting practices resulting from preterm birth. Future research should further investigate the underlying mechanisms.

The observation that relationship difficulties primarily stem from initiating rather than maintaining relationships highlights VP/VLBW individuals'‐specific characteristics and needs and has important implications for intervention strategies. Early intervention programs focusing on social skills development during childhood and adolescence could be particularly beneficial, with emphasis on initiating conversations and building initial connections. Although research has consistently shown that children born preterm have poorer social skills [8], are more likely to be bullied [9], and social difficulties are a major concern for parents, surprisingly no studies have attempted to improve social skills in this group of children and young people. Furthermore, supportive programs during key life transitions, such as entering school, changes in educational levels, entering the workforce or navigating new social environments, could be especially valuable. Given that there is still a lack of awareness among healthcare providers and educators about the long‐term consequences of preterm birth, having schemes and educational programs such as the Preterm Birth Information for Educational Professionals and the Prem Aware Award (UK), would help foster tailored support to these children. Dating applications or social networking platforms specifically designed for individuals who find initial social contact challenging could be worth exploring, though careful consideration would need to be given to avoiding stigmatisation.

Several questions remain unanswered. While preterm children's intellectual and cognitive development has been extensively studied, their social development has received less attention. Little is known about preterm children's socio‐cognitive skills, such as Theory of Mind. Research has shown that preterm children have reduced joint attention compared to their full‐term peers [10], being less likely to initiate joint attention with others and respond to others' engagement efforts. This early impairment may represent the first step in a cascade of maladjusted social development that requires investigation.

Furthermore, large‐observational studies are needed to identify protective factors that enable VP/VLBW individuals to develop social networks despite early challenges. This requires collaborative research and the utilisation of infrastructures such as the RECAP preterm platform, which hosts the world's largest and most comprehensive collection of longitudinal data from birth into childhood and adulthood of VP/VLBW individuals from European VP birth cohorts and Nordic registers. This line of research should examine a comprehensive range of resilience factors, including individual, family, social and educational influences. Understanding these resilience factors will be essential to inform effective interventions.

Finally, the impact of modern neonatal care practices on long‐term social outcomes remains unclear. Given that the current study's participants were born in the 1980s, research must examine whether contemporary neonatal care practices, including increased parent–infant contact (e.g. Kangaroo Care) and family‐centred care, influence long‐term social outcomes. Recent research showing positive outcomes from early interventions, such as music therapy in neonatal intensive care units [11], suggests promising directions for future practice. However, careful evaluation of these approaches' long‐term impact on social development is essential.

The persistent nature of social relationship differences into established adulthood, coupled with the identification of specific challenges in relationship initiation, provides clear direction for future research and intervention development.

As survival rates of VP/VLBW infants continue to improve, understanding and supporting their long‐term social development becomes increasingly critical. The resilience observed in parent–child relationships provides an encouraging foundation for building other social connections, while the persistent challenges in peer and partner relationships highlight the need for continued support. Future research should focus on understanding mechanisms underlying social difficulties, developing and evaluating interventions focused on social skills, particularly targeted support for relationship initiation while being careful to avoid approaches that might increase stigmatisation. Additionally, identifying protective factors and understanding the impact of modern neonatal care practices will be crucial for informing effective interventions and improving the long‐term social trajectories of individuals born very preterm.

## Author Contributions

M. Mendonça is solely responsible for the contents of this commentary.

## Conflicts of Interest

The author declares no conflicts of interest.

## Data Availability

The author has nothing to report.
